# Single-channel properties of α3β4, α3β4α5 and α3β4β2 nicotinic acetylcholine receptors in mice lacking specific nicotinic acetylcholine receptor subunits

**DOI:** 10.1113/jphysiol.2012.246595

**Published:** 2013-04-22

**Authors:** Anna Ciuraszkiewicz, Wolfgang Schreibmayer, Dieter Platzer, Avi Orr-Urtreger, Petra Scholze, Sigismund Huck

**Affiliations:** 1 Division of Pathobiology of the Nervous System, Center for Brain Research, Medical University of Vienna Spitalgasse 4, A-1090 Vienna, Austria; 2 Institute for Biophysics, Center of Physiological Medicine, Medical University of Graz Harrachgasse 21/IV, A-8010 Graz, Austria; 3 Genetic Institute, Tel-Aviv Sourasky Medical Center and Sackler School of Medicine, Tel Aviv University 6 Weizmann Street, Tel Aviv 64239, Israel

## Abstract

Previous attempts to measure the functional properties of recombinant nicotinic acetylcholine receptors (nAChRs) composed of known receptor subunits have yielded conflicting results. The use of knockout mice that lack α5, β2, α5β2 or α5β2α7 nAChR subunits enabled us to measure the single-channel properties of distinct α3β4, α3β4α5 and α3β4β2 receptors in superior cervical ganglion (SCG) neurons. Using this approach, we found that α3β4 receptors had a principal conductance level of 32.6 ± 0.8 pS (mean ± SEM) and both higher and lower secondary conductance levels. α3β4α5 receptors had the same conductance as α3β4 receptors, but differed from α3β4 receptors by having an increased channel open time and increased burst duration. By contrast, α3β4β2 receptors differed from α3β4 and α3β4α5 receptors by having a significantly smaller conductance level (13.6 ± 0.5 pS). After dissecting the single-channel properties of these receptors using our knockout models, we then identified these properties – and hence the receptors themselves – in wild-type SCG neurons. This study is the first to identify the single-channel properties of distinct neuronal nicotinic receptors in their native environment.

**Key points**

- Under normal conditions, individual nerve cells express a cohort of several nicotinic acetylcholine receptor (nAChR) subtypes with unique functional properties. Therefore, single- channel recordings of such neurons reflect the mixed properties of the various expressed receptors.
- Previous attempts to recapitulate the properties of naturally occurring receptors using recombinant receptors of known subunit composition in heterologous expression systems have been largely unsuccessful, as the properties of these receptors vary widely among expression systems.
- We measured the properties of specific nAChRs in superior cervical ganglion neurons cultured from mice with targeted deletions of select nAChR subunit genes. Mice lacking both the α5 and the β2 subunits express α3β4 receptors, whereas single-knockout (KO) mice lacking either α5 or β2 express α3β4β2 and α3β4α5 (plus α3β4) hetero-oligomeric receptors, respectively. This approach allows one to investigate these receptors at the single-channel level in their native environment. The single-channel properties of nACh receptors in superior cervical ganglion (SCG) neurons from α5β2α7 triple-KO mice were similar to the properties of receptors measured in α5β2 double-KO mice.
- The principal conductance level of α3β4 receptors was 32.6 ± 0.8 pS (mean ± SEM), and these receptors also displayed both higher and lower secondary conductance levels. The conductance levels of α3β4α5 receptors were identical to α3β4 receptors, but the α3β4α5 receptors had longer open times and burst duration. α3β4β2 receptors had a lower conductance level and longer open times than α3β4 receptors. Interestingly, all three receptor types could be identified faithfully in wild-type C57Bl/6J SCG neurons.

## Introduction

Nicotinic acetylcholine receptors (nAChRs) are homopentameric or heteropentameric ligand-gated ion channels containing five identical or different subunits, respectively. The predominant hetero-oligomeric nAChR in the CNS contains α4β2 subunits, whereas α3β4 is the most prevalent channel in the peripheral nervous system (McGehee & Role, 1995). However, the presence of additional subunits throughout virtually the entire nervous system can potentially give rise to a dazzling variety of receptors that probably differ in their pharmacological and biophysical properties (McGehee & Role, 1995; Gotti *et al.* 2006). Unlike the neuromuscular junction, in which the receptors contain a defined subunit composition, the properties of nAChRs in individual nerve cells are largely unknown. Hence, the properties of specific neuronal receptors have traditionally been investigated using either heterologous expression systems such as *Xenopus laevis* oocytes and HEK293 cells or neuroblastoma cell lines such as IMR-32 and SH-SY5Y cells (Nelson & Lindstrom, 1999; Nelson *et al.* 2001). However, the properties of these recombinant receptors may not accurately reflect the properties of receptors in their native environment or – even worse – may depend on the host cell type (Papke, 1993; Lewis *et al.* 1997; Sivilotti *et al.* 1997).

To reduce the variety of nAChR subunits and thereby bypass these problems, some groups have knocked down the expression of specific subunits using antisense oligonucleotides (Listerud *et al.* 1991; Brussaard *et al.* 1994; Yu & Role, 1998*a*). The sympathetic nervous system is particularly well suited to this approach, as it contains a relatively small cohort of intrinsic receptor subunits (Listerud *et al.* 1991). Although such knockdown experiments have yielded important information, they require the prior removal of surface receptors (by the covalent binding of bromoacetylcholine), leaving some ambiguity regarding the subunit composition of the receptors that are actually expressed.

Here, we took a different approach, recording superior cervical ganglion (SCG) neurons from mice that have a genetic deletion (knockout) of their α5 and/or β2 nAChR genes, thereby resulting in the expression of α3β4 nAChRs with or without the β2 (i.e. α3β4 and α3β4β2) or α5 (i.e. α3β4 and α3β4α5) subunit (David *et al.* 2010). Our results represent the first measurements of the single-channel properties of ‘pure’α3β4 nAChRs in their native environment and reveal the contributions of the α5 and β2 subunits to the functional properties of these channels. We found that the addition of the α5 subunit to α3β4 channels had a prominent effect on channel open time and burst duration, whereas the addition of the β2 subunit significantly decreased unitary conductance. The properties of these receptors measured in the knockout strains were then identified in SCG neurons cultured from wild-type (WT) C57Bl/6J mice.

## Methods

### Ethics approval

Animals were housed and all experiments were performed in accordance with the European Communities Council directive (86/609/EEC) and the Austrian federal law governing animal experimentation (Tierversuchsgesetz TVG 501/1989).

### Animals

Experiments were performed using cultured neurons isolated from the SCG of WT C57Bl/6J mice or from mice carrying genetic deletions of the genes encoding the nAChR subunits α5 (Wang *et al.* 2002), β2 (Picciotto *et al.* 1995) or α7 (Orr-Urtreger *et al.* 1997). β2 knockout (KO) mice were generously provided by J.-P. Changeux (Pasteur Institute, Paris, France), and α7 KO mice were purchased from Jackson Laboratories (Bar Harbor, ME, USA). Mice lacking both the α5 and the β2 subunits (α5β2 double-KO) were obtained by crossing the two respective single-KO lines. The α5α7β2 triple-KO mice were obtained by crossing α5β2 double-KO mice with α7 single-KO animals. The mice used in this study were backcrossed onto the C57Bl/6J background for seven (α5, α7) or 12 (β2) generations after germ-line transmission. All animals were housed in a climate-controlled room (at 21°C) with a light–dark schedule of 10/14 h in group cages with free access to food and water.

### Cell culture of SCG neurons

The SCG were dissected from 5–6-day-old (P5–P6) mouse pups following decapitation. The enzymes, trituration protocol and culture conditions were similar to published procedures (Fischer *et al.* 2005), with the addition of 10% fetal calf serum (FCS; Sigma F7524, St Louis, MO, USA) to the culture medium during trituration. We seeded 10,000 dispersed cells on laminin-coated 35 mm tissue culture dishes (Thermo Scientific-Nunc, Waltham, MA, USA), and the cells were cultured in 5% CO_2_ at 36.5°C for 3–5 days before use.

### Solutions and reagents

The bath and pipette solution was as follows (in mm): 120 NaCl, 3 KCl, 2 MgCl_2_, 2 CaCl_2_, 20 glucose, 10 Hepes, 0.1 μm atropine and 0.5 μm tetrodotoxin (TTX; Latoxan, Valence, France), adjusted to pH 7.3 with NaOH. All of the recordings shown in our figures were performed with 3 μm ACh, and most of the data presented in Table 1 were collected with 3 μm ACh (38 out of 43 patches from the α5β2 and α5β2α7 KO neurons; 11/16 patches from α5 KO neurons; 13/17 patches from β2 KO neurons; and all patches from WT neurons). For the remaining patches, the pipette solution contained 5 μm ACh. These ACh concentrations are well below the EC_50_ as determined by whole-cell recordings (Kristufek *et al.* 1999; Fischer *et al.* 2005; David *et al.* 2010). We did not observe any nAChR channel activity in the absence of ACh (*n*= 17 patches from α5β2 KO neurons). Furthermore, a total of 86 patches showed no channel openings even in the presence of ACh and presumably contained no channels. ACh concentrations ranging from 1 to 10 μm have mostly been used for recording the single-channel activity of both heterologously expressed α3β4 receptors and endogenous nAChRs in the SCG (Papke & Heinemann, 1991; Mathie *et al.* 1991; Lewis *et al.* 1997; Nelson & Lindstrom, 1999; Krashia *et al.* 2010). The general chemical reagents used in our experiments were obtained from Merck-VWR (Radnor, PA, USA); unless otherwise specified, the remaining substances were obtained from Sigma- Aldrich (St. Louis, MO, USA).

**Table 1:** Summary of main slope conductance levels and kinetic parameters of nAChRs in wild-type (WT) and nAChR subunit knockout SCG neurons

	α5β2 +α5β2α7 KO	β2 KO		α5 KO		WT		
				
Genotype Receptor	α3β4	α3β4	α3β4α5 α3β4	α3β4	α3β4β2	α3β4	α3β4α5 α3β4	α3β4β2
Main conductance (pS)	32.6 ± 0.8 (*n*= 32)^1^	32.9 ± 1.2 (*n*= 13)^5^		32.8 ± 0.9 (*n*= 7/16)^9^	13.6 ± 0.5 (*n*= 7/16)^13^	33.7 ± 0.9 (*n*= 23)^15^	32.5 ± 1.9 (*n*= 6/23)^15^	15.4 ± 0.8 (*n*= 4/23)
	α5β2 double-KO 32.1 ± 0.8 (*n*= 17)^2^	32.1 ± 2.9 (*n*= 5)^6^	33.5 ± 1.1 (*n*= 8)^7^	32.3 ± 1.3 (*n*= 9/16)^10^				
	α5β2α7 triple-KO 33.3 ± 1.5 (*n*= 15)^3^							
τ open time (ms)	τ1: 0.79 ± 0.10 τ2: 8.99 ± 0.55 (*n*= 43) 11a	τ1: 0.65 ± 0.09 τ2: 8.75 ± 0.94 (*n*= 9/17)	τ1: 0.30 ± 0.09 τ2: 9.16 ± 0.85 τ3: 56.7 ± 7.2 (*n*= 8/17)^7^	τ1: 1.17 ± 0.25 τ2: 11.2 ± 1.5 (*n*= 7/16)^11^	τ1: 1.27 ± 0.59 τ2: 25.4 ± 3.9 (*n*= 7/16)	τ1: 0.89 ± 0.23 τ2: 13.4 ± 1.4 (*n*= 17/23)	τ1: 0.38 ± 0.07 τ2: 7.63 ± 1.32 τ3: 46.3 ± 7.2 (*n*= 6/23)	τ1: 0.67 ± 0.05 τ2: 19.0 ± 1.7 (*n*= 4/23)
	α5β2 double-KO τ1: 0.81 ± 0.14 τ2: 9.64 ± 0.70 (*n*= 25)^2^			τ1: 0.78 ± 0.19 τ2: 10.0 ± 0.9 (*n*= 9/16)^12^				
	α5β2α7 triple-KO τ1: 0.77 ± 0.13 τ2: 8.10 ± 0.85 (*n*= 18)^3^							
τ burst length (ms) median (25th, 75th percentile)	τ: 27.3 (18.0, 32.4) (*n*= 43)^1,4^	τ: 27.5 (22.0, 39.6) (*n*= 9/17)	τ1: 25.4 (16.1, 45.5) τ2: 151.9 (104.5, 215.3) (*n*= 8/17)^8^	τ: 32.3 (23.9, 54.3) (*n*= 7/16)^11^	n.d.^14^	τ: 38.5 (30.3, 55.3) (*n*= 16/23)	τ1: 27.9 (16.1, 47.6) (*n*= 4/23)^16^ τ2: 115.9 (60.1, 275.9) (*n*= 6/23)^16^	n.d.^14^
	α5β2 double-KO τ: 29.1 (19.1, 34.0) (*n*= 25)^2,4^			τ: 47.4 (36.6, 63.0)				
	α5β2α7 triple-KO τ: 22.6 (16.8, 31.9) (*n*= 18)^3,4^							

1 Pooled data from α5β2 double-KO and α5β2α7 triple-KO mice.

1a Open times in all patches could be fitted using the sum of two exponential components, although three exponential components yielded a good fit in some patches as well.

2 Data from α5β2 double-KO mice.

3 Data from α5β2α7 triple-KO mice. Burst durations in α5β2 double-KO and α5β2α7 triple-KO mice do not differ significantly (*P* > 0.05, Mann–Whitney test).

4 Median values. Burst durations calculated upon separation of bursts by critical closed times (as described in the Methods section) were not normally distributed (*P* < 0.001^1,2^, Kolmogorov–Smirnov test).

5 α3β4 and α3β4α5 receptors have the same conductance: eight of these patches contained channels with extra-long openings (τ3: 56.7 ± 7.2 ms). The slope conductance of these putative α3β4α5 channels was 33.5 ± 1.1 pS, not significantly different from α3β4 channels in α5β2/α5β2α7 KO neurons^1^ (32.6 ± 0.8 pS, *n*= 32; *P* > 0.05, Student's *t* test).

6 The open time p.d.f. was fitted best by a double-exponential function with the time constants τ1 = 0.63 ± 0.14 (27 ± 8%) and τ2 = 8.89 ± 1.40 (73 ± 8%; *n*= 5).

7 The open time p.d.f. was fitted best by a triple-exponential function in eight of the β2 KO patches. Thus, τ3 was attributed to α3β4α5 receptors.

8 Burst duration in which channel open times required a three-exponential fit.

9 Channel conductance in patches in which low-conductance channels were also present.

10 Channel conductance in patches in which low-conductance channels were not present.

11 Kinetics in patches in which low-conductance channels were present. Burst durations do not significantly differ from bursts in α5β2/α5β2α7 KO neurons (*P* > 0.05, Mann–Whitney test).

12 Kinetics in patches in which low-conductance channels were not present. Burst durations differ significantly from bursts in α5β2/α5β2α7 KO neurons (*P* < 0.05, Mann–Whitney test).

13 Significantly different from conductance in α5β2/α5β2α7 KO (*P* > 0.0001, Student's *t* test).

14 Not determined due to the rarity of bursts.

15 All 23 patches taken from WT animals showed channel activity with a mean conductance of 33.7 pS. In six of these patches, the channels had extra-long openings and extended bursts with a mean conductance of 32.5 pS. Four patches contained channels with markedly reduced conductance and longer openings.

16 Two of the patches required fit to a single-exponential function.

^1,5,9,15^ Conductances do not differ significantly among the genotypes (*P* > 0.05, one-way ANOVA).

*n*= number of patches with the indicated channel activity. Unless indicated otherwise, data are mean values ± SEM.

### Single-channel patch-clamp recordings

Single-channel currents were amplified, filtered, recorded and analysed using an Axopatch 200B amplifier, a Digidata 1320A digitizer/data acquisition system and pClamp 10 software (all from Axon/Molecular Devices, Sunnyvale, CA, USA). All recordings were performed in the cell-attached patch configuration (Hamill *et al.* 1981), which minimizes ‘run down’ of channel activity, a problem that is often encountered with excised (outside-out) patches (Papke, 1993; Lewis *et al.* 1997). Patch pipettes were fabricated from thick-walled borosilicate glass (1.5 mm × 0.86 mm; Science Products), and the tips were coated with Sylgard 184 (Dow Corning) and fire-polished to a final resistance of 8–17 MΩ when filled with pipette solution. All experiments were performed at room temperature (21–23°C). We recorded at least two continuous minutes of activity; the data were low-pass filtered at 5 kHz using the amplifier's four-pole Bessel filter, sampled at 50 kHz and stored on the computer's hard disk.

### Analysis

The recordings were digitally filtered at 3.5 kHz (8-pole Bessel filter, –3 dB) and converted by visual control into event lists based on a 50% threshold criterion using Clampfit's single-channel search function. Events lasting longer than 1.3 times the filter rise time (*T*_r_) were used for kinetic analyses (Colquhoun & Sigworth, 1983). The filter rise time was calculated as follows: *T*_r_= 0.3321/*f*_c_, where *f*_c_ is the effective cut-off frequency of the system (Colquhoun & Sigworth, 1983). The event lists were imported into MATLAB software (version 7.8.0, The Mathworks, Inc., Natick, MA, USA) using routines as described previously (Yakubovich *et al.* 2000). Any portion of a record in which two or more channels opened simultaneously was excluded from the analysis. Amplitude histograms were generated for events (i.e. openings) with a duration that was longer than twice the filter rise time (*T*_r_). The amplitude distribution histograms were fitted with the appropriate number of Gaussian components using the maximum-likelihood method. Single-channel (i.e. unitary) conductance was estimated from the slope of a linear fit of the mean current amplitude measured at pipette potentials of 30, 50, 70 and 90 mV, and kinetics were analysed as described previously (Yakubovich *et al.* 2000).

We typically analysed the kinetic properties of the channels at a pipette potential of 70 mV. However, due to their lower conductance, the open times of the α3β4β2 receptors (in the α5 KO mice) were analysed at a pipette potential of 90 mV. Because calculations of slope conductance require channel activity recorded over a range of patch potentials, the number of patches in which the conductance could be determined was usually fewer than the number of patches that were analysed for open and closed times and/or burst durations.

**Figure 1:**
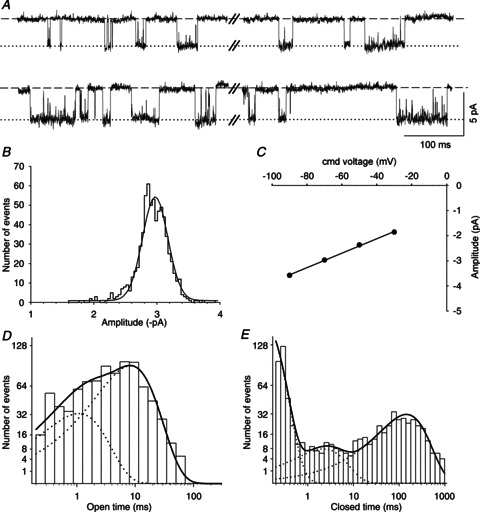
Conductance and kinetic properties of α3β4 nAChRs *A*, exemplar segments of a continouous cell-attached recording of single-channel activity from an SCG neuron cultured from an α5β2α7 triple-KO mouse. Channel openings were induced at a patch pipette potential of 70 mV by the addition of 3 μm ACh to the pipette solution. In this and subsequent figures, the dashed lines indicate the closed (zero-conductance) state, and the dotted lines indicate the open state of the channel. *B*, amplitude histogram generated as described in the Methods section and Gaussian fit (continuous line) from a 2 min recording at a patch pipette potential of 70 mV (mean amplitude ± SEM: −2.97 ± 0.01 pA). *C*, *I–V* plot: command (cmd) voltage plotted against the amplitude obtained from amplitude histograms generated at patch pipette potentials of 90, 70, 50 and 30 mV. The error bars (SEM) are smaller than the symbols and are therefore not visible. The calculated slope conductance is 28.8 ± 0.8 pS. *D*, channel open time distribution fitted by the sum of two exponential components (continuous line) with time constants τ1 = 1.07 ms (23% of all events) and τ2 = 8.32 ms (77% of all events). These data could also be fitted by the sum of three exponential components with time constants τ1 = 0.45 ms (10% of all events), τ2 = 4.89 ms (57% of all events) and τ3 = 11.9 ms (33% of all events). *E*, channel closed time distribution fitted with the sum of three exponential components with time constants τ1 = 0.11 ms, τ2 = 2.21 ms and τ3 = 144.4 ms (with relative frequencies of 40, 11 and 49%, respectively). The data in *B–E* were derived from the patch shown in *A*.

Probability density distributions of the open and closed times were fitted to a probability density function (p.d.f.) with *N* exponential terms as follows:


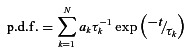
(1)

where *a_k_* (a weighting factor) is the contribution of the *k*th exponential component to the total area under the fitted curve, *t* is time and τ*_k_* is the characteristic time constant of the *k*th component (Colquhoun & Hawkes, 1995). When pooling the data obtained from the α5β2 and α5β2α7 KO mice, the closed times were fitted best by a triple-exponential function with time constants τ1 0.24 ± 0.03 ms (32 ± 2% of all events), τ2 11.9 ± 2.9 ms (20 ± 1% of all events) and τ3 1794 ± 271 ms (48 ± 2% of all events; *n*= 43; see Fig. 1*E* for an example). Closed times in the rat SCG recorded at low concentrations of ACh (≤5 μm) were reportedly best fitted by the sum of four exponential components (35.9 μs, 0.73 ms, 39.7 ms and 1704 ms), although three exponential components were sufficient in some cases (Mathie *et al.* 1991). For reasons discussed in this paper, measuring open and closed times was more difficult for rat SCG neurons than for nicotinic channels in frog and rat muscle endplates. Because closed times are the basis for calculating the critical closed time (*t*_crit_), this problem carries over to the analysis of bursts. Parameter *t*_crit_ defines the time interval that separates channel closures within a burst from closures between bursts. From the several possible ways to assess *t*_crit_ (discussed in Colquhoun & Sakmann, 1985, but see also Derkach *et al.* 1987 and Papke & Heinemann, 1991), we chose the model proposed by Colquhoun & Sakmann (1985):


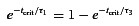
(2)

To obtain a clear separation of bursts, we inserted τ1 and τ3 into the equation (ratios τ3/τ1 consistently > 100). The mean critical closed time for the pooled data obtained from the α5β2 and α5β2α7 KO neurons calculated using this method was 1.77 ± 0.22 ms (*n*= 43). In 41 out of 43 patches, the individual *t*_crit_ assessed for each patch was shorter than its corresponding τ2, meaning that in 95% of the patches τ2 appeared as a closed interval that separated the burst events. So τ1 evidently represents the closed times within bursts, but the more obvious choice for insertion into eqn (2) might have been τ2 rather τ3 (as closed times that separated the bursts). The *t*_crit_ calculated using this method was 0.71 ± 0.12 ms, and the burst duration was moderately shortened (pooled data from α5β2 double-KO and α5β2α7 triple-KO neurons (*n*= 43 neurons): median: 22.8 ms; 25th percentile: 16.6 ms; 75th percentile: 31.0 ms). However, choosing τ1 and τ2 resulted in a significant overlap (and thus misclassifaction) of closures within and between bursts (τ2/τ1 ratios averaged from 43 patches: 44.6 ± 6.8, which is well below the preferred ratio of 100; see Edmonds *et al.* (1995)).

The burst length distribution was then calculated and is presented as described above for open and closed times (Table 1). The burst duration distributions of both muscle-type and neuronal nAChRs are usually fitted with a double exponential function, with the fast time constant representing a single apparent opening event (Colquhoun & Sakmann, 1985; Derkach *et al.* 1987; Mathie *et al.* 1991; Papke & Heinemann, 1991). Because our MATLAB routine for fitting burst duration was programmed to ignore single openings, the bursts recorded from α3β4 receptors were fitted best with a single-exponential function (median τ: 27.3 ms). However, as discussed above (Mathie *et al.* 1991) we cannot exclude the possibility that our single openings contain brief closed events that could not be resolved in our analysis.

### Statistics

Unless stated otherwise, all summary data are presented as mean ± SEM. Kolmogorov–Smirnov normality, Student's unpaired *t* test, non-parametric Mann–Whitney test or one-way ANOVA were performed where appropriate using GraphPad Prism 5.04.

## Results

### Single-channel properties of α3β4 receptors

SCG neurons from α5β2 double-KO mice express a single class of heteropentameric nAChRs containing only α3 and β4 subunits (David *et al.* 2010). Hence, channels recorded from these neurons will not be contaminated by the presence of α5 or β2 subunits, each of which is incorporated into approximately 25% of nACh receptors in WT animals (David *et al.* 2010).

**Figure 2:**
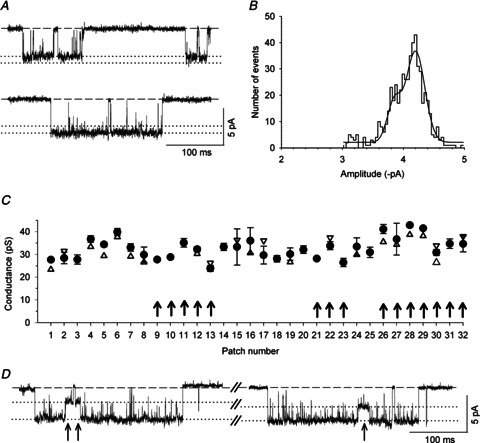
Subconductance states of α3β4 receptors *A*, exemplar segments of a continouous cell-attached recording of single-channel activity at a patch pipette potential of 70 mV from an α5β2 double-KO SCG neuron; these segments were selected to show the subconductance levels. The secondary conductance level in the upper panel was observed at a sufficient frequency to become evident as a shoulder in the amplitude histogram. *B*, amplitude histogram and Gaussian fits from a 2 min recording of the patch shown in *A*. The shoulder at −3.85 ± 0.03 pA reflects a secondary conductance level that was slightly lower than the main conductance state (at −4.19 ± 0.02 pA). These data were derived from the patch shown in *A*. *C*, summary of the channel conductances recorded from 32 separate patches. The filled circles represent the main conductance level ± SEM, and the triangles show the lower (up triangles) and higher (down triangles) secondary conductance levels. The arrows indicate recordings made from α5β2α7 triple-KO neurons. *D*, exemplar segments of a continouous cell-attached recording at a patch pipette potential of 70 mV from an α5β2 double-KO SCG neuron; these segments were selected to show transitions to a secondary conductance state that always occurred during the main state (arrows). This secondary conductance state was too rare to become evident in the amplitude histogram (*B*) and is therefore not shown in *C*.

Figure 1*A–C* shows original exemplar recordings, the amplitude histogram at a patch pipette potential of 70 mV and the *I–V* plot obtained from an SCG neuron recorded in the cell-attached configuration. The recordings – as well as the amplitude histogram in Fig. 1*B*– show that α3β4 receptors appear to have only one conductance level. However, channels recorded from 21 out of 32 patches had less frequent secondary conductance levels that were reflected by different current amplitudes both in the original recordings and in the amplitude histogram (Fig. 2*A* and *B*). These secondary conductance levels were smaller than the principal conductance in 14 patches and larger in six patches. One patch displayed both smaller and larger secondary conductances, in addition to the main conductance level (Fig. 2*C*). The actual percentage of patches with these secondary conductance levels was probably higher, as secondary conductances that were identified in the original traces but were not evident in the amplitude histograms – due either to their relative rarity or to having an amplitude close to the principal conductance level – were not included. The prevailing (i.e. principal) conductance level was 32.6 ± 0.8 pS (*n*= 32 patches, Table 1).

Channel openings with an amplitude that differed from the main conductance level were observed both in isolation (Fig. 2*A*) and in conjunction with the predominant channel activity (Fig. 2*D*). Hence, channels with lower conductance (approximately 50% of the main conductance) that transitioned directly to the main conductance state as shown in Fig. 2*D* were rare and were present in only four out of 32 patches. In contrast, the β2-containing channels described below never transitioned directly to the main conductance level. Direct transitions from the main conductance level to a secondary conductance level suggest the presence of a single receptor with different conductive states (Mathie *et al.* 1991; Sivilotti *et al.* 1997). However, secondary conductance levels that occur in isolation could be due to the presence of more than one receptor in the patch. Indeed, we often observed superimposed channel openings that were seen as a doubling of the main conductance level (data not shown).

We next analysed these channels’ open times, closed times and burst durations at a pipette potential of 70 mV. The open time p.d.f. was generally fitted best by a double-exponential function with time constants of 0.79 ± 0.10 and 8.99 ± 0.55 ms (representing 28 ± 3 and 72 ± 3% of all events, respectively; *n*= 43 patches; Table 1; see Fig. 1*D* for an example).

Rat SCG neurons express homopentameric α7-containing receptors (Cuevas *et al.* 2000). We therefore considered the possibility that some of the channels recorded in the α5β2 double-KO mice might have been α7 homopentameric receptors. However, adding 5 nm methyllycaconitine to the pipette solution had no effect (*P* > 0.05; these data were analysed using Student's *t* test, except for burst duration, which was analysed using the Mann–Whitney test) on any of the following parameters: conductance: 32.3 ± 1.2 pS; open time τ1: 0.50 ± 0.18 ms; τ2: 8.52 ± 0.75 ms; τ burst duration: 26.2 ms (25th percentile: 23.6; 75th percentile: 36.3); *n*= 7 patches. These values were not different from values measured in the absence of methyllycaconitine: conductance: 32.1 ± 0.8 pS (*n*= 17); open time τ1: 0.81 ± 0.14 ms; τ2: 9.64 ± 0.70 ms; τ burst duration: 29.1 ms (*n*= 25 patches; see Table 1). Furthermore, all of the parameters were similar between channels recorded from α5β2 double-KO neurons and α5β2α7 triple-KO neurons (Fig. 2*C*, Table 1); therefore, we pooled the data collected from these two genotypes.

### Single-channel properties of α3β4α5 receptors in the β2 single-KO mice

We next investigated whether the added presence of the α5 subunit modifies the properties of the α3β4 receptor channel by recording SCG neurons from β2 single-KO mice. Using immunoprecipitation and Western blot analyses, we previously found that the SCG neurons of β2 KO mice express two populations of nicotinic receptors, namely α3β4 (76%) and α3β4α5 (24%) (David *et al.* 2010). Assuming that these receptor populations are also present at the cell surface, we can expect to record channel activity from either α3β4 or α3β4α5 receptors.

**Figure 3:**
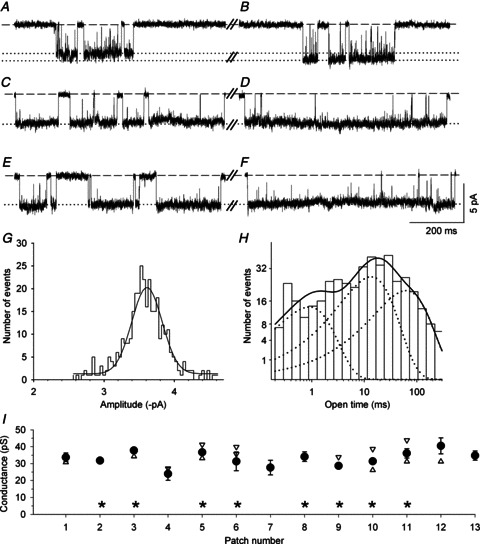
Conductance and kinetic properties of nAChRs in the β2 KO mouse model *A* and *B*, exemplar segments of a continouous cell-attached recording of single-channel activity from a β2 KO SCG neuron. These recordings are similar to the traces shown in Fig. 1*A* and Fig. 2A for the α5β2/α5β2α7 KO neurons, both with respect to their frequent closings and amplitudes. *C–F*, exemplar segments recorded from the same patch in *A* and *B*, showing channel activity with extra-long openings that are not interupted by closures and presumably result from the added presence of the α5 subunit (see *H* and Fig. 4*B*, *C*). *G*, amplitude histogram and Gaussian fit from a 2 min recording at a patch pipette potential of 70 mV (mean amplitude ± SEM: −3.61 ± 0.01 pA). *H*, channel open time distribution fitted with the sum of three exponential components (continuous line) with time constants τ1 = 0.89 ms (18% of all events), τ2 = 13.5 ms (46% of all events) and τ3 = 61.9 ms (36% of all events). The data from *G* and *H* were derived from the patch shown in *A–F*. *I*, summary of the channel conductance levels recorded from 13 different patches. The filled circles represent the main conductance level (±SEM), and the triangles show the lower (up triangles) and higher (down triangles) secondary conductance levels. The asterisks indicate the eight patches in which the channel open time distributions required fitting by three exponential components with pooled time constants of 0.30 ± 0.09 ms (10 ± 2% of all events), 9.16 ± 0.85 ms (61 ± 5% of all events) and 56.7 ± 7.2 ms (29 ± 4% of all events).

Interestingly, the channel conductances measured in the β2 KO neurons were nearly identical to the levels measured in the α5β2/α5β2α7 KO neurons (main slope conductance: 32.9 ± 1.2 pS, *n*= 13 patches, *P* > 0.05, Student's *t* test; Fig. 3*A–G*, *I*; Table 1). However, in 8 of the 17 patches that were analysed for channel kinetics, openings of notably longer duration were identified in addition to the channels that were observed in the α5β2/α5β2α7 KO neurons (Fig. 3*C–F*). The open time p.d.f. in patches with these novel channels was fitted best by three exponential components with time constants of 0.30 ± 0.09 ms (10 ± 2% of all events), 9.16 ± 0.85 ms (61 ± 5% of all events) and 56.7 ± 7.2 ms (29 ± 4% of all events; see Table 1 for a summary and Fig. 3*H* for an example). We therefore conclude that α5-containing receptors favour prolonged channel openings and that these openings require a third exponential for fitting the p.d.f. The slope conductance of these channels was 33.5 ± 1.1 pS (*n*= 8), which was not significantly different from the α3β4 receptors recorded from the α5β2/α5β2α7 KO neurons (*P* > 0.05, Student's *t* test). However, in 9 of the 17 patches that were recorded from β2 single-KO neurons, the p.d.f. of the open times could be fitted best by a double-exponential function that yielded results that were nearly identical to the channels recorded from the α5β2/α5β2α7 KO neurons (with time constants of 0.65 ± 0.09 and 8.75 ± 0.94 ms, representing 24 ± 5 and 76 ± 5% of all events, respectively; Table 1). Therefore, these patches were clearly devoid of significant activity by α3β4α5 receptors.

**Figure 4:**
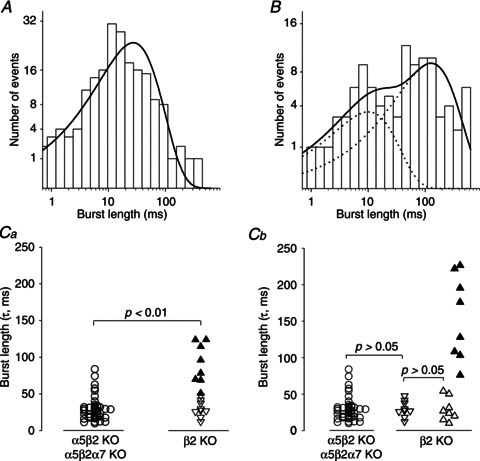
Burst length analysis in α5β2 double-KO (and α5β2α7 triple-KO) patches *versus*β2 single-KO patches *A*, channel burst length distribution of α3β4 nAChRs recorded from the same patch shown in Fig. 1. The burst length distribution was fitted with one exponential component with a time constant of 27.3 ms. *B*, channel burst length distribution of nAChRs recorded from a β2 single-KO neuron (the same patch shown in Fig. 3*A–H*). The burst length distribution was fitted with two exponential components with time constants of 10.1 and 127.8 ms. *Ca*, summary of the burst durations of α3β4α5 receptors (recorded from β2 single-KO neurons) and α3β4 receptors (recorded from pooled α5β2/α5β2α7 double/triple-KO neurons). The p.d.f. of each burst length distribution was fitted to a single exponential in both the α5β2/α5β2α7 (circles) and the β2 KO neurons (triangles). The burst durations in the α5β2/α5β2α7 KO neurons were not distributed normally (*P* < 0.001, Kolmogorov–Smirnov test) and had a median τ of 27.3 ms (25th percentile: 18.0; 75th percentile: 32.4; *n*= 43 patches). The filled triangles show the burst length distributions from β2 single-KO patches that had extra-long channel openings and required fitting of their open time p.d.f. with a triple-exponential function (see Table 1 and Fig. 3*H* for an example). In contrast, the open triangles show the burst length distributions from β2 single-KO patches that had channel openings that were fitted to a double-exponential function. The burst durations differed significantly (*P* < 0.01, Mann–Whitney test) between the α5β2/α5β2α7 KO (median: 27.3 ms; *n*= 43) and β2 single-KO neurons (median: 47.6 ms; 27.2 and 87.3 for the 25th and 75th percentiles, respectively; *n*= 17). *Cb*, burst length distributions from the β2 KO neurons were resolved into three elements. The down triangles show the burst durations that were fitted with one exponential (median: 27.5 ms; 22.0 and 39.6 for the 25th and 75th percentiles, respectively; *n*= 9) and open times that were fitted with two exponential components. The up triangles show the burst durations that were fitted with two exponentials (open triangles: τ1 median: 25.4 ms; 16.1 and 45.5 for the 25th and 75th percentiles, respectively; *n*= 8; filled triangles: τ2 median 151.9 ms; 104.5 and 215.3 for the 25th and 75th percentiles, respectively; *n*= 8) and open times that were fitted with three exponential components. Statistical comparisons were made using the Mann–Whitney test.

When expressed in *X. laevis* oocytes, α3β4α5 receptors have significantly longer burst durations than α3β4 receptors (Nelson & Lindstrom, 1999). It was therefore particularly interesting to compare the burst length between β2 single-KO and the pooled α5β2/α5β2α7 double/triple-KO neurons. Bursts in α5β2/α5β2α7 KO neurons were best fitted with a single-exponential function (Fig. 4*A*). Most of these bursts had durations that clustered below 40 ms. However, 6 out of 43 patches had longer bursts, causing a skewed distribution of burst times (*P* < 0.001, Kolmogorov–Smirnov test). We therefore treated the burst times in all of the genotypes under the assumption that their distributions might also not have a Gaussian distribution. The median burst duration of the α3β4 receptors was 27.3 ms (*n*= 43 patches; Fig. 4*Ca*, Table 1), and the mean (±SEM) number of channel openings per burst was 3.5 ± 0.2.

When fitted with a single-exponential function, burst duration as a whole was significantly longer in the β2 KO neurons than in the α5β2/α5β2α7 KO neurons (β2 single-KO median: 47.6 ms; 25th percentile: 27.2; 75th percentile: 87.3; *n*= 17; *P* < 0.01, Mann–Whitney test; Fig. 4*Ca*). Moreover, the burst durations in patches recorded from β2 KO neurons fell into two discrete populations that were linked to the channel open times. When the p.d.f. of channel open times was fitted using two exponential components, the corresponding median burst duration was 27.5 ms (25th percentile: 22.0; 75th percentile: 39.6; *n*= 9 patches; Fig. 4*Ca* and *Cb*, Table 1), similar to results obtained in α5β2/α5β2α7 KO neurons (Fig. 4*Cb*). In contrast, when open time p.d.f. was fitted using three exponentials (Fig. 3*H*), median burst duration when fitted to a single exponential was increased significantly to 87.3 ms (25th percentile: 69.1; 75th percentile: 121.4; *n*= 8 patches; *P* < 0.0001, Mann–Whitney test; Fig. 4*Ca*, filled triangles). Because we expect two populations of receptors in this cohort, burst durations may be fitted better by a double-exponential function (Fig. 4*B*), with median time constants τ1: 25.4 ms (Fig. 4*Cb*, open up triangles) and τ2: 151.9 ms (*n*= 8; Fig. 4*Cb*, filled triangles; Table 1). Thus, τ1 and τ2 correspond to the burst durations of α3β4 and α3β4α5 receptors, respectively. Because the number of openings per burst did not differ significantly between the two burst lengths (4.1 ± 0.3 openings per long burst, *n*= 8 patches *vs.* 3.7 ± 0.2 openings per ‘regular’ burst, *n*= 9 patches; indicated by up and down triangles, respectively, in Fig. 4*Ca*; *P* > 0.05, Student's *t* test), we conclude that in the β2 KO neurons, the extra-long channel openings caused by the presence of the α5 subunit also account for the longer burst duration.

### Single-channel properties of α3β4β2 receptors in α5 single-KO mice

**Figure 5:**
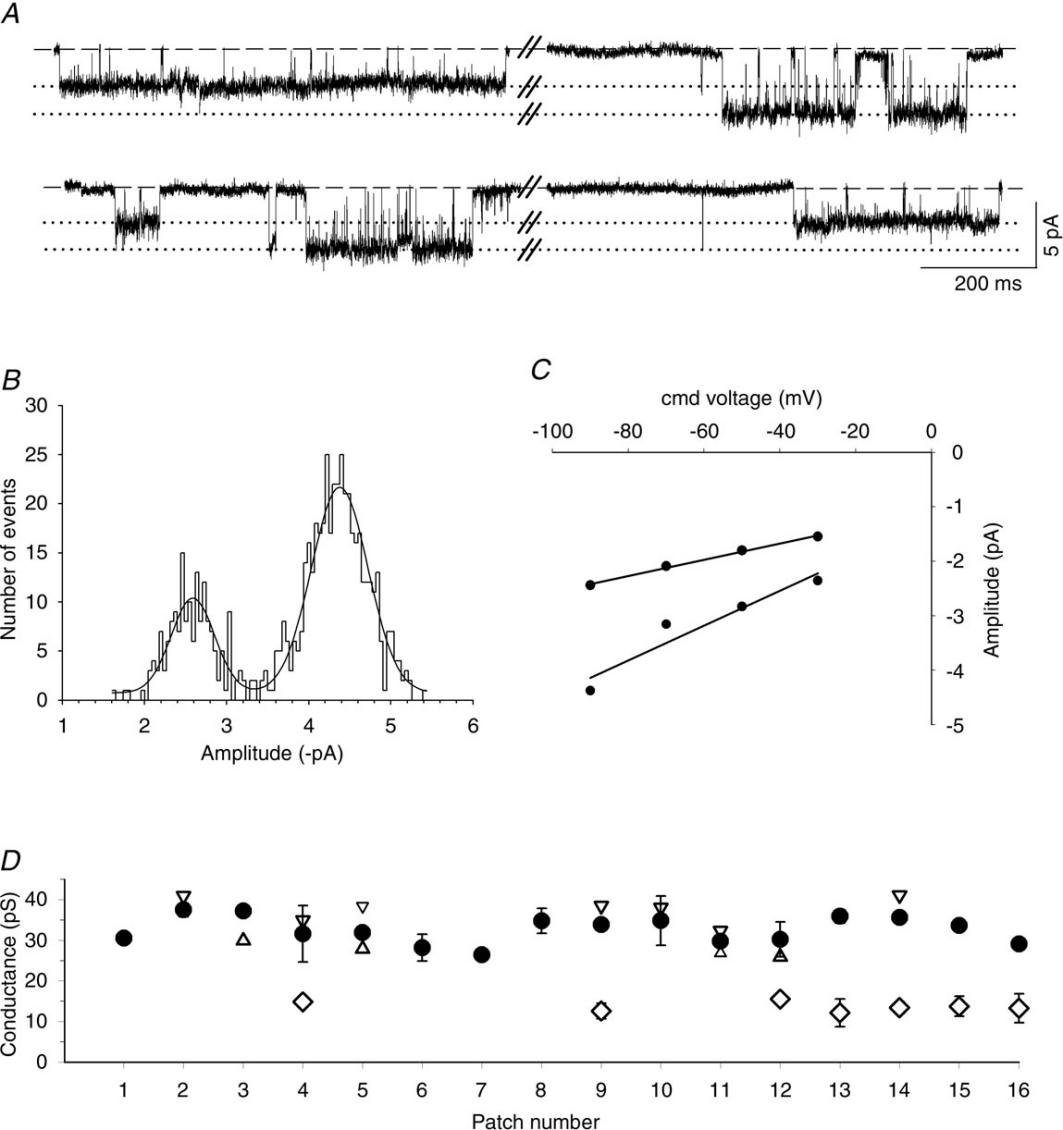
Conductance and kinetic properties of channels recorded in α5 KO neurons *A*, exemplar segments of a continouous cell-attached recording of single-channel activity from an α5 KO SCG neuron at a patch pipette potential of 90 mV. Note the prominent channel openings with amplitudes that differed by approximately 50%. *B*, amplitude histogram and Gaussian fit from a 2 min recording at a patch pipette potential of 90 mV showing two discrete peaks at –2.44 ± 0.01 and −4.37 ± 0.02 pA. *C*, *I–V* plot generated from the amplitude histograms at patch electrode potentials of 30, 50, 70 and 90 mV. The calculated slope conductances were 14.8 ± 0.8 and 31.6 ± 6.9 pS. *D*, summary of channel conductance levels from 16 patches. The circles represent the main conductance levels (±SEM), and the triangles show the lower (up triangles) and higher (down triangles) secondary conductance levels. The diamonds show a significantly smaller conductance level (±SEM) that was observed only in α5 KO patches. Note that these channels always occur together with – but did not transition directly between – receptors that are normally found in α5β2 double-KO neurons. For a summary of the kinetic parameters, see Table 1.

nAChRs in the SCG neurons of α5 single-KO mice consist of 79%α3β4 and 21%α3β4β2 receptors (David *et al.* 2010). We can therefore expect to find two populations of surface receptors in these neurons, one population comprising α3β4 receptors and one population comprising α3β4β2 receptors. In 7 of the 16 patches that were recorded from α5 single-KO neurons, we indeed identified receptors with properties that differed from the typical α3β4 receptors described above. These channels had a lower conductance level (13.6 ± 0.5 pS; *n*= 7) combined with somewhat longer open times and occurred prominently and in isolation (Fig. 5, Table 1). The open time distribution was fitted best by two exponential components with time constants of 1.27 ± 0.59 ms (19 ± 3% of all events) and 25.4 ± 3.9 ms (81 ± 3% of all events, *n*= 7 patches). We attribute these properties to α3β4β2 receptors. In addition, in these patches we always observed channel activity that was similar to the α3β4 receptors, with a main conductance level of 32.8 ± 0.9 pS (*n*= 7 patches, Fig. 5, Table 1). Nine of the 16 patches in the α5 single-KO neurons exhibited channel activity that lacked the characteristics of β2-containing receptors, with a main conductance level of 32.3 ± 1.3 pS (Fig. 5*D*) and open times that were fitted best by two exponential components (0.78 ± 0.19 and 10.0 ± 0.9 ms; Table 1). Thus, these channels are clearly α3β4 receptors.

### Single-channel properties of nAChRs in WT mice

**Figure 6:**
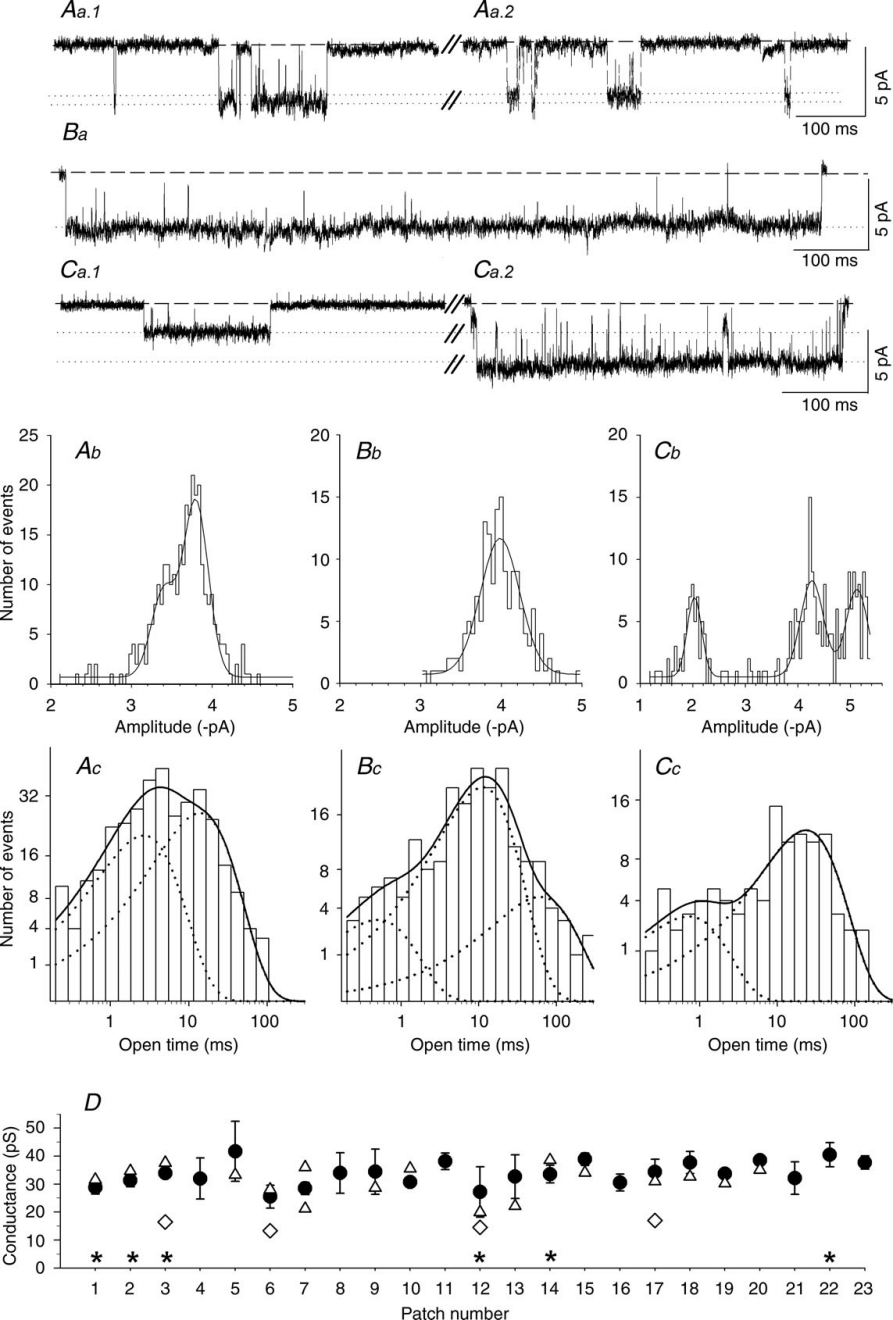
Conductance and kinetic properties of nACh receptors in wild type SCG neurons *Aa–Ca*, exemplar segments of continouous cell-attached recordings of single-channel activity from SCG neurons cultured from three different WT mice. Channel openings were recorded at a patch pipette potential of either 70 mV (*Aa1*, *Aa2* and *Ba*) or 90 mV (*Ca1* and *Ca2*). *Aa1*, the amplitude (slope conductance: 33 pS, data not shown) and relatively brief openings indicate the presence of an α3β4 receptor. *Aa2*, channel activity in the same patch as *Aa1* showing a slightly smaller conductance level (see *Ab*). *Ba*, the amplitude (slope conductance: 31 pS, data not shown) and relatively long openings that are not interrupted by channel ‘flickering’ suggest the presence of an α3β4α5 receptor. *Ca1*, the amplitude (−2.01 ± 0.02 pA, see *Cb*) and somewhat longer openings (see *Cc*) suggest the presence of an α3β4β2 receptor. *Ca2*, the amplitude (−5.11 ± 0.02 pA, see *Cb*) and relatively brief openings indicate the presence of an α3β4 receptor in the same patch. *Ab–Cb*, amplitude histograms and Gaussian fits from 2 min segments of the recodings shown in traces *Aa–Ca*. *Ab*, main peak: −3.80 ± 0.01 pA; secondary peak: −3.40 ± 0.03 pA. *Bb*, single peak at −3.98 ± 0.01 pA. *Cb*, channel openings at three different amplitudes (−2.01 ± 0.02, −4.26 ± 0.02 and −5.11 ± 0.02 pA) were revealed in this patch. *Ac–Cc*, channel open time distributions from 2 min segments of the recordings shown in *Aa–Ca*. *Ac*, the data were fitted with the sum of two exponential components (continuous line) with τ1 = 1.71 ms (38% of all events) and τ2 = 15.0 ms (62% of all events). *Bc*, the data were fitted best with the sum of three exponential components (continuous line): τ1 = 0.50 ms (7% of all events), τ2 = 11.2 ms (74% of all events) and τ3 = 61.3 ms (19% of all events). *Cc*, kinetic analysis of the low-conductance state; data were fitted best with the sum of two exponential components (continuous line): τ1 = 0.72 (15% of all events) and τ2 = 23.3 ms (85% of all events). *D*, summary of the channel conductances measured from 23 patches. The circles represent the main slope conductances (±SEM), and the triangles show the secondary conductance levels. The diamonds show nAChRs in patches that – based on their conductance and longer open times – have the characteristics of α3β4β2 channels (15.4 ± 0.8 pS; τ1 = 0.67 ± 0.05 ms, τ2: 19.0 ± 1.7 ms; means ± SEM, *n*= 4). The asterisks indicate the six patches that contained putative α3β4α5 receptors (mean conductance ± SEM: 32.5 ± 1.9 pS) for which the channel open-time distributions required fitting with three exponential components with time constants τ1 = 0.38 ± 0.07 ms (7 ± 2% of all events), τ2 = 7.63 ± 1.32 ms (59 ± 10% of all events) and τ3 = 46.3 ± 7.2 ms (34 ± 10% of all events).

Because SCG neurons in WT mice express three types of nAChRs, namely α3β4 (55%), α3β4α5 (24%) and α3β4β2 (21%) (David *et al.* 2010), we would expect a rather complex mixture of receptor channels that have disparate properties. Given the information obtained from recording receptors from the various KO mice, we tested whether these distinct receptors could be detected in WT SCG neurons on the basis of their single-channel properties (Fig. 6).

We analysed 23 WT patches, all of which displayed the high conductance level (33.7 ± 0.9 pS; Fig. 6*D*, Table 1), in most cases in addition to secondary conductance levels (Fig. 6*Aa*, *Ab*, *D*). Six of the 23 patches displayed a high conductance level (32.5 ± 1.9 pS) in conjunction with long bursts (τ2 median: 115.9 ms) and prominent long openings (which required the p.d.f. of the open time distribution to be fitted with three exponential components as follows: τ1: 0.38 ± 0.07 ms, 7 ± 2%; τ2: 7.63 ± 1.32 ms, 59 ± 10%; τ3: 46.3 ± 7.2 ms, 34 ± 10%; Table 1). Based on the properties described above, this class of receptors probably represents α3β4α5 channels (Fig. 6*Ba*, *Bc*, *D*). Putative α3β4β2 receptors were identified in 4 of the 23 patches (Fig. 6*Ca1*, *Cb*, *Cc*, *D*) and are characterized by their lower conductance (15.4 ± 0.8 pS) combined with prolonged openings (τ1: 0.67 ± 0.05 ms; τ2: 19.0 ± 1.7 ms; Table 1). The actual frequency of patches containing α3β4β2 or α3β4α5 receptors in WT SCGs is likely to be higher, as we included such channels in our analysis only when they occurred frequently enough to be recognized in the amplitude histogram (α3β4β2) or in the exponential fit of the open time p.d.f. (α3β4α5). Patches displaying neither low-conductance (i.e. 15.4 pS) nor high-conductance (e.g. 32.5 pS) channels with extra-long openings appear to have contained primarily α3β4 receptors (Fig. 6*Aa*, *Ab*, *Ac*, *Ca2*, *D*).

## Discussion

The goal of this study was to investigate the single-channel properties of discrete hetero-oligomeric nAChRs containing specific receptor subunits in their native cellular environment. We chose the SCG as our model system because this structure expresses a limited and previously identified cohort of nAChR subunits. The subunit composition of hetero-oligomeric nAChRs is similar between rats (Mao *et al.* 2006) and mice, with a distribution of approximately 55%α3β4, 24%α3β4α5 and 21%α3β4β2 receptors in the mouse SCG (David *et al.* 2010). Furthermore, mouse KO models are available that express pure populations of α3β4 receptors (in α5β2 double-KO mice), α3β4 and α3β4β2 receptors (in α5 single-KO mice), and α3β4 and α3β4α5 receptors (in β2 single-KO mice) (David *et al.* 2010). We reasoned that these KO models would allow us to investigate the properties of α3β4 receptors as well as how the addition of the α5 and β2 subunits would modulate their function. The intrinsic variety of receptor types that are normally expressed in the SCG has always precluded the analysis of individual receptor types in this structure (Derkach *et al.* 1987; Mathie *et al.* 1991; Sivilotti *et al.* 1997; Lewis *et al.* 1997).

Because of the mRNA species that have been identified in the SCG, the α7, α3 and β4 subunits are expected to comprise the majority of receptors, followed by the α5 and β2 subunits (Mandelzys *et al.* 1994; Putz *et al.* 2008). The presence of two types of functional α7 nAChRs has been suggested in the rat SCG (Cuevas *et al.* 2000), and experiments based on an α7 oligonucleotide antisense strategy together with the application of the α7-specific nAChR antagonists methyllycaconitine and α-bungarotoxin indicate that α7-containing receptors are expressed in chick sympathetic neurons (Yu & Role, 1998*b*). However, in the present study, channel activity did not differ significantly between receptors that were recorded in patches from α5β2 double-KO and α5β2α7 triple-KO neurons, and methyllycaconitine did not affect the single-channel properties of receptors in α5β2 double-KO neurons, suggesting that α7-containing receptors were not measurably activated under our experimental conditions. Consistent with this notion, we previously reported that whole-cell currents recorded from cultured mouse SCG neurons contained α7 nAChR currents only in the presence of the α7 allosteric modulator PNU-120596 (David *et al.* 2010).

The α3 and β4 subunits are prevalent in the rat SCG, and recombinant α3β4 receptors (for example, expressed in *X. laevis* oocytes) should have properties similar to native receptors in the rat SCG. Unexpectedly, however, neither recombinant α3β4 nor recombinant α3β4α5 receptors resemble native receptors recorded in rat SCG neurons (Sivilotti *et al.* 1997). Moreover, the channel properties of recombinant α3β4 receptors vary depending on the heterologous system in which they are expressed (Lewis *et al.* 1997; Sivilotti *et al.* 1997). We therefore revisited native receptors in the SCG by recording nACh receptors that contain a known subunit composition, which was achieved by the targeted genetic deletion of select nAChR subunit genes (David *et al.* 2010).

### α3β4 receptors

We found that α3β4 receptors (i.e. recorded from α5β2 double-KO neurons) have an average main conductance level of 32.6 pS, which is quite similar to human α3β4 receptors expressed heterologously in either *X. laevis* oocytes or HEK293 cells (31 pS, Nelson & Lindstrom, 1999; Nelson *et al.* 2001). Likewise, nAChRs in IMR-32 human neuroblastoma cells – which contain primarily α3 and β4 subunits – have a conductance level of 32 pS (Nelson & Lindstrom, 1999). Although most of the patches containing rat α3β4 receptors expressed in BOSC 23 cells had a mean conductance level of 34 pS (Ragozzino *et al.* 1997), the primary conductance level of rat α3β4 receptors was 22 pS when expressed in oocytes (Papke & Heinemann, 1991; Sivilotti *et al.* 1997), and the conductance was either high (i.e. 30–40 pS) or low (i.e. 20–26 pS) when these receptors were stably transfected into L-929 mouse fibroblasts (Lewis *et al.* 1997). The authors suggested that these seemingly diverse populations of receptors might represent different folding and/or assembly of the subunits, different stoichiometry and/or different post-translational modifications. Lewis *et al.* (1997) concluded that ‘whatever the reasons may be, the practical consequences are that neither oocytes nor L-929 cells can be relied on to produce a homogenous population of channels when transfected with two subunit types’.

In addition to a main conductance level, the majority of our patches also exhibited channel openings that were less frequent and carried either slightly larger or smaller (secondary) conductance levels. In most cases, these states were separated by channel closings, which may reflect the presence of more than one channel in the patch (Mathie *et al.* 1991). In mice lacking both α5 and β2 subunits, we can exclude the possibility that either of these subunits accounts for the secondary conductance states in these neurons. It is also unlikely that alternative stoichiometry underlies these differences in channel conductance in mouse SCG neurons, as the conductance levels of human α3β4 receptors expressed in HEK293 cells differ by as much as 33% when expressed at an α/β ratio of either 9:1 or 1:9 (Krashia *et al.* 2010). Thus, the rather subtle variations in α3β4 channel conductance that we observed might be due to post-translational regulation of receptors, interactions with chaperone proteins and/or cytoskelatal components, or membrane microenvironments (Nelson & Lindstrom, 1999; Barrantes, 2004; Albuquerque *et al.* 2009). It is also worth mentioning that in early postnatal mice, α3 mRNA levels are approximately 2-fold higher than β4 mRNA levels (Putz *et al.* 2008), whereas in rats, β4 mRNA is approximately 1.5-fold higher than α3 mRNA at birth, although the relative mRNA levels reverse by postnatal day 14 (Mandelzys *et al.* 1994).

### α3β4α5 receptors

The channels recorded from patches in β2 single-KO SCG neurons differed from α5β2 double-KO neurons by having noticeably longer openings (reflected by an additional τ3 of 56.7 ms) and increased burst duration (when fitted to a double-exponential function, the pooled median time constant τ2 for burst duration was 151.9 ms). Similar effects on channel kinetics were observed when rat α5 subunits were expressed together with α3β4 receptors in *X. laevis* oocytes; specifically, the open time distributions of these receptors revealed a novel, slower component (22 ms), and burst duration increased from 16 to 43 ms (Nelson & Lindstrom, 1999). However, in contrast to our results (which showed no differences in conductance levels between β2 single-KO and α5β2 double-KO neurons), the addition of α5 subunits to α3β4 receptors in oocytes resulted in additional, moderately increased conductance levels (Sivilotti *et al.* 1997; Nelson & Lindstrom, 1999).

### α3β4β2 receptors

Channels recorded from patches in α5 single-KO SCG neurons differed from α5β2 double-KO neurons by having a significantly lower conductance level (13.6 pS) and open times that were approximately 2.5-fold longer in duration (τ2: 25.4 ms). This type of channel activity was always recorded together with receptors that matched the properties of α3β4 receptors, suggesting preferential co-localization of these two receptors in the same patch. We never observed a direct transition from 13.6 pS (i.e. β2-containing) receptors to the more common 32.6 pS α3β4 receptor channels. Our results differ from previous results obtained using *X. laevis* oocytes, in which the conductance level (22 pS) was identical regardless of whether α3β4 receptors were expressed in isolation or together with β2 subunits (Sivilotti *et al.* 1997). Native α3β4β2 receptors are particularly interesting due to their presence both in the rodent (rat and mouse) SCG (Mao *et al.* 2006; David *et al.* 2010) and in the mouse habenula (Scholze *et al.* 2012).

### nAChRs in WT animals

After we established the functional properties of native α3β4 receptor channels and the impact of adding the α5 or β2 subunit, we next attempted to identify these channels in SCG neurons of WT mice. As shown in Fig. 6, all receptor types could be identifed and had properties that were in good agreement with the results that we obtained from the KO models (Table 1). In WT neurons, the predominant channels had a conductance level of 33.7 pS and kinetics that were similar to the α3β4 receptors that we recorded in the pooled α5β2/α5β2α7 double/triple-KO neurons. We also observed putative α3β4α5 and α3β4β2 receptors based on their distinctive kinetics and lower conductance, respectively.

Previous single-channel nAChR recordings in rat SCG neurons displayed a wide range of mean main conductance levels, including 20 pS (in 4-week-old animals; 50 pS channels were observed at a lesser frequency; Derkach *et al.* 1987), 34.8 pS (in adult animals; Sivilotti *et al.* 1997), 36.6 pS (in 1-day-old animals; Sivilotti *et al.* 1997) to 36.8 pS (in 17- to 21-day-old animals; Mathie *et al.* 1991). Our data therefore match quite closely those obtained by Mathie *et al.* (1991) and Sivilotti *et al.* (1997). Interestingly, a smaller conductance of 15.3 pS has also been observed in adult rat SCG patches by Sivilotti *et al.* (1997). Based on our comparison between α5β2 double-KO and α5 single-KO mice, we attribute this conductance to α3β4β2 receptors. Minor disparities among these results might be due to different preparations (e.g. SCG neurons from P5–P6 mice cultured for 3–5 days *vs.* acutely dissociated cells from rats of various ages), differences in the recording solutions and/or different methods of analysing the data. For example, our bath solution contained 2 mm Ca^2+^ and 2 mm Mg^2+^, whereas Colquhoun's group used 1 mm Ca^2+^ and 2 mm Mg^2+^ (Mathie *et al.* 1991; Sivilotti *et al.* 1997; Lewis *et al.* 1997). The external solution used by Derkach *et al.* (1987) contained 2.5 mm Ca^2+^ and 1.2 mm Mg^2+^. Indeed, divalent cations are known to affect nAChR conduction; omitting calcium from the bath solution enhanced single-channel conductance in rat SCG neurons from 36.8 to 51 pS (Mathie *et al.* 1991), whereas raising the calcium concentration inhibited the conductance of nAChRs in PC12 cells with an IC_50_ of 6.9 mm (Neuhaus & Cachelin, 1990). The disparity among these results highlights the fact that using identical conditions to record and analyse channels is essential when attempting to directly compare ion channel properties (see Lewis *et al.* 1997, for further discussion).

### nAChR channel kinetics determine the properties of synaptic currents

The time course of a postsynaptic current is the sum of the time courses of all individual single-channel currents through each receptor in the postsynaptic membrane. If the neurotransmitter is available for binding to the postsynaptic receptors for a short duration of time (as is the case at the neuromuscular junction), an individual receptor is less likely to activate repeatedly during a single synaptic event (Edmonds *et al.* 1995; Wyllie *et al.* 1998). There is general consensus that a single receptor activation event (i.e. an event that occurs while the agonist is bound to the receptor) causes oscillations (called a burst) between the open state (A2R*) and short-lived closed states (A2R) (Colquhoun & Sakmann, 1985; Sine & Steinbach, 1987; Papke, 1993; Edmonds *et al.* 1995; Wyllie *et al.* 1998). The properties of these bursts can be analysed if the likelihood of re-association between the ligand and receptor is low (e.g. with a low steady-state agonist concentration or immediately following a rapid jump to zero concentration) (Edmonds *et al.* 1995). The presence of a high concentration of agonist will increase the occurrence of bursts and will introduce additional non-conducting periods due to channel block and – if the high concentration is sustained – receptor desensitization (Colquhoun & Sakmann, 1985; Sine & Steinbach, 1987; Papke, 1993). Assuming that the receptors will activate synchronously and then deactivate at random intervals (due to near instantaneous agonist binding and stochastic agonist unbinding, respectively), the decay in synaptic current should therefore reflect the average rate at which the channels close (Colquhoun & Sakmann, 1985; Papke, 1993; Edmonds *et al.* 1995; Skok, 2002; Hatton *et al.* 2003). With respect to the neuromuscular junction, predictions derived from steady-state single-channel recordings have turned out to be reasonably accurate (Edmonds *et al.* 1995; Elenes *et al.* 2006). In addition, an empirical confirmation of reaction schemes to determine synaptic events was obtained by the application of a brief (i.e. lasting only a few milliseconds), rapid stepwise pulse application of agonist (Edmonds *et al.* 1995; Wyllie *et al.* 1998; Elenes *et al.* 2006). Hence, although most experimental studies examined only the decay phase of macroscopic currents in response to such a jump in agonist concentration, their results are generally in good agreement with the deactivation properties of bursts measured from single-channel recordings at low agonist concentrations (Wyllie *et al.* 1998; Elenes *et al.* 2006).

Unfortunately, the statement by Edmond *et al*. (1995) that far less is known about neuronal nicotinic receptors and synaptic currents still holds true. In sympathetic ganglia, the following excitatory postsynaptic current decay time constants have been recorded: 4.2 ms (recorded in lower lumbar ganglia at resting membrane potential and 37°C, Hirst & McLachlan, 1984), 13.9 ms (recorded in the rat SCG at −110 mV and 23°C, Derkach *et al.* 1987) and 16 ms (recorded in the rabbit SCG at −80 mV and 34–37°C, Derkach *et al.* 1983). However, the decay of postsynaptic currents varies with both temperature (Magleby & Stevens, 1972*a*; Rang, 1981; Hirst & McLachlan, 1984) and membrane potential (Magleby & Stevens, 1972*b*; Rang, 1981; Derkach *et al.* 1983). Similarly, membrane potential also affects the kinetics of muscle-type nAChRs (assessed by single-channel recordings, Mishina *et al.* 1986) and neuronal nAChRs (assessed by relaxation kinetics, Figl & Cohen, 2000), precluding comparisons between synaptic (i.e. macroscopic) nAChR currents and single-channel nAChR properties unless performed under similar conditions. Excitatory postsynaptic currents in the (parasympathetic) rat submandibular ganglion decay with two voltage-dependent time constants, τ1 (5–9 ms) and τ2 (27–45 ms) (recorded at membrane potentials ranging from −30 to −100 mV and at 20°C, Rang, 1981). Because the vast majority of nAChRs that are expressed in the autonomic nervous system are of the α3β4* type (Mao *et al.* 2006), these two time constants may reflect the (slow) open time and the burst duration that we measured for α3β4 receptors in the mouse SCG (τ_2open_: 8.99 ms; τ_burst_: 27.3 ms). The significantly longer burst duration of α3β4α5 receptors relative to α3β4 receptors argues against the preferential clustering of these receptors at SCG synapses. Synaptic clustering of α5-containing receptors has been proposed for mouse SCG neurons that have been transformed into the cholinergic phenotype by culturing in the presence of ciliary neurotrophic factor (Gingras *et al.* 2002). However, because subunit-specific antibodies are not available, obtaining immunohistochemical data to confirm these fíndings is not currently feasible.

### Summary

We utilized mice that carry genetic deletions in their α5, α7 and/or β2 nAChR subunits and therefore express distinct α3β4, α3β4α5 and α3β4β2 receptors. Our novel results reveal the properties of α3β4 receptors in their native environment. Specifically, α5-containing receptors have longer open times and burst durations, whereas β2-containing receptors have a channel conductance that is approximately half the conductance of α3β4 (and α3β4α5) receptors. We found that α5- and β2-containing receptors never occurred in isolation but always together with α3β4 receptors. Having assessed the profiles of α3β4, α3β4α5 and α3β4β2 receptors in neurons from the respective KO mice, we then identified these receptors in WT SCG neurons.

## Abbreviations

KO: knockout
nAChR: nicotinic acetylcholine receptor
p.d.f.: probability density function
SCG: superior cervical ganglion
WT: wild-type

## References

[b1] Albuquerque EX, Pereira EFR, Alkondon M, Rogers SW (2009). Mammalian nicotinic acetylcholine receptors: from structure to function. Physiol Rev.

[b2] Barrantes FJ (2004). Structural basis for lipid modulation of nicotinic acetylcholine receptor function. Brain Res Brain Res Rev.

[b3] Brussaard AB, Yang X, Doyle JP, Huck S, Role LW (1994). Developmental regulation of multiple nicotinic AChR channel subtypes in embryonic chick habenula neurons: contribution of both the α2 and α4 subunit genes. Pflügers Arch.

[b4] Colquhoun D, Hawkes AG, Sakmann B, Neher E (1995). The priniciples of the stochastic interpretation of ion channel mechanisms. *Single-channel Recording*.

[b5] Colquhoun D, Sakmann B (1985). Fast events in single- channel currents activated by acetylcholine and its analogues at the frog muscle end-plate. J Physiol.

[b6] Colquhoun D, Sigworth FJ, Sakmann B, Neher E (1983). Fitting and statistical analysis of single channel records. *Single-channel Recording*.

[b7] Cuevas J, Roth AL, Berg DK (2000). Two distinct classes of functional α7-containing nicotinic receptor on rat superior cervical ganglion neurons. J Physiol.

[b8] David R, Ciuraszkiewicz A, Simeone X, Orr-Urtreger A, Papke RL, McIntosh JM, Huck S, Scholze P (2010). Biochemical and functional properties of distinct nicotinic acetylcholine receptors in the superior cervical ganglion of mice with targeted deletions of nAChR subunit genes. Eur J Neurosci.

[b9] Derkach VA, North RA, Selyanko AA, Skok VI (1987). Single channels activated by acetylcholine in rat superior cervical ganglion. J Physiol.

[b10] Derkach VA, Selyanko AA, Skok VI (1983). Acetylcholine- induced current fluctuations and fast excitatory post-synaptic currents in rabbit sympathetic neurones. J Physiol.

[b11] Edmonds B, Gibb AJ, Colquhoun D (1995). Mechanisms of activation of muscle nicotinic acetylcholine receptors and the time course of endplate currents. Annu Rev Physiol.

[b12] Elenes S, Ni Y, Cymes GD, Grosman C (2006). Desensitization contributes to the synaptic response of gain-of-function mutants of the muscle nicotinic receptor. J Gen Physiol.

[b13] Figl A, Cohen BN (2000). The β subunit dominates the relaxation kinetics of heteromeric neuronal nicotinic receptors. J Physiol.

[b14] Fischer H, Orr-Urtreger A, Role LW, Huck S (2005). Selective deletion of the α5 subunit differentially affects somatic–dendritic *versus* axonally targeted nicotinic ACh receptors in mouse. J Physiol.

[b15] Gingras J, Rassadi S, Cooper E, Ferns M (2002). Agrin plays an organizing role in the formation of sympathetic synapses. J Cell Biol.

[b16] Gotti C, Zoli M, Clementi F (2006). Brain nicotinic acetylcholine receptors: native subtypes and their relevance. Trends Pharmacol Sci.

[b17] Hamill OP, Marty A, Neher E, Sakmann B, Sigworth FJ (1981). Improved patch-clamp techniques for high-resolution current recording from cells and cell-free membrane patches. Pflugers Arch.

[b18] Hatton CJ, Shelley C, Brydson M, Beeson D, Colquhoun D (2003). Properties of the human muscle nicotinic receptor, and of the slow-channel myasthenic syndrome mutant ɛL221F, inferred from maximum likelihood fits. J Physiol.

[b19] Hirst GD, McLachlan EM (1984). Post-natal development of ganglia in the lower lumbar sympathetic chain of the rat. J Physiol.

[b20] Krashia P, Moroni M, Broadbent S, Hofmann G, Kracun S, Beato M, Groot-Kormelink PJ, Sivilotti LG (2010). Human α3β4 neuronal nicotinic receptors show different stoichiometry if they are expressed in *Xenopus* oocytes or mammalian HEK293 cells. PLoS One.

[b21] Kristufek D, Stocker E, Boehm S, Huck S (1999). Somatic and prejunctional nicotinic receptors in cultured rat sympathetic neurones show different agonist profiles. J Physiol.

[b22] Lewis TM, Harkness PC, Sivilotti LG, Colquhoun D, Millar NS (1997). The ion channel properties of rat recombinant neuronal nicotinic receptor are dependent on the host cell type. J Physiol.

[b23] Listerud M, Brussaard AB, Devay P, Colman DR, Role LW (1991). Functional contribution of neuronal AChR subunits revealed by antisense oligonucleotides. Science.

[b24] Magleby KL, Stevens CF (1972a). A quantitative description of end-plate currents. J Physiol.

[b25] Magleby KL, Stevens CF (1972b). The effect of voltage on the time course of end-plate currents. J Physiol.

[b26] Mandelzys A, Pie B, Deneris ES, Cooper E (1994). The developmental increase in ACh current densities on rat sympathetic neurons correlates with changes in nicotinic ACh receptor subunit gene expression and occurs independent of innervation. J Neurosci.

[b27] Mao D, Yasuda RP, Fan H, Wolfe BB, Kellar KJ (2006). Heterogeneity of nicotinic cholinergic receptors in rat superior cervical and nodosa ganglia. Mol Pharmacol.

[b28] Mathie A, Cull-Candy SG, Colquhoun D (1991). Conductance and kinetic properties of single nicotinic acetylcholine receptor channels in rat sympathetic neurones. J Physiol.

[b29] McGehee DS, Role LW (1995). Physiological diversity of nicotinic acetylcholine receptors expressed by vertebrate neurons. Annu Rev Physiol.

[b30] Mishina M, Takai T, Imoto K, Noda M, Takahashi T, Numa S, Methfessel C, Sakmann B (1986). Molecular distinction between fetal and adult forms of muscle acetylcholine receptor. Nature.

[b31] Nelson ME, Lindstrom J (1999). Single channel properties of human α3 AChRs: impact of β2, β4 and α5 subunits. J Physiol.

[b32] Nelson ME, Wang F, Kuryatov A, Choi CH, Gerzanich V, Lindstrom J (2001). Functional properties of human nicotinic AChRs expressed by IMR-32 neuroblastoma cells resemble those of α3β4 AChRs expressed in permanently transfected HEK cells. J Gen Physiol.

[b33] Neuhaus R, Cachelin AB (1990). Changes in the conductance of the neuronal nicotinic acetylcholine receptor channel induced by magnesium. Proc Biol Sci.

[b34] Orr-Urtreger A, Göldner FM, Saeki M, Lorenzo I, Goldberg L, De Biasi M, Dani JA, Patrick JW, Beaudet AL (1997). Mice deficient in the α7 neuronal nicotinic acetylcholine receptor lack α-bungarotoxin binding sites and hippocampal fast nicotinic currents. J Neurosci.

[b35] Papke RL (1993). The kinetic properties of neuronal nicotinic receptors: genetic basis of functional diversity. Progr Neurobiol.

[b36] Papke RL, Heinemann SF (1991). The role of β4-subunit in determining the kinetic properties of rat neuronal nicotinic acetylcholine α3-receptors. J Physiol.

[b37] Picciotto MR, Zoli M, Lena C, Bessis A, Lallemand Y, Le Novere N, Vincent P, Pich EM, Brulet P, Changeux J-P (1995). Abnormal avoidance learning in mice lacking functional high-affinity nicotine receptor in the brain. Nature.

[b38] Putz G, Kristufek D, Orr-Urtreger A, Changeux JP, Huck S, Scholze P (2008). Nicotinic acetylcholine receptor-subunit mRNAs in the mouse superior cervical ganglion are regulated by development but not by deletion of distinct subunit genes. J Neurosci Res.

[b39] Ragozzino D, Fucile S, Giovanelli A, Grassi F, Mileo AM, Ballivet M, Alema S, Eusebi F (1997). Functional properties of neuronal nicotinic acetylcholine receptor channels expressed in transfected human cells. Eur J Neurosci.

[b40] Rang HP (1981). The characteristics of synaptic currents and responses to acetylcholine of rat submandibular ganglion cells. J Physiol.

[b41] Scholze P, Koth G, Orr-Urtreger A, Huck S (2012). Subunit composition of α5-containing nicotinic receptors in the rodent habenula. J Neurochem.

[b42] Sine SM, Steinbach JH (1987). Activation of acetylcholine receptors on clonal mammalian BC3H-1 cells by high concentrations of agonist. J Physiol.

[b43] Sivilotti LG, McNeil DK, Lewis TM, Nassar MA, Schoepfer R, Colquhoun D (1997). Recombinant nicotinic receptors, expressed in *Xenopus* oocytes, do not resemble native rat sympathetic ganglion receptors in single-channel behaviour. J Physiol.

[b44] Skok VI (2002). Nicotinic acetylcholine receptors in autonomic ganglia. Autonom Neurosci.

[b45] Wang N, Orr-Urtreger A, Chapman J, Rabinowitz R, Nachmann R, Korczyn AD (2002). Autonomic function in mice lacking α5 neuronal nicotinic acetylcholine receptor subunit. J Physiol.

[b46] Wyllie DJ, Behe P, Colquhoun D (1998). Single-channel activations and concentration jumps: comparison of recombinant NR1a/NR2A and NR1a/NR2D NMDA receptors. J Physiol.

[b47] Yakubovich D, Pastushenko V, Bitler A, Dessauer CW, Dascal N (2000). Slow modal gating of single G protein-activated K^+^ channels expressed in *Xenopus* oocytes. J Physiol.

[b48] Yu CR, Role LW (1998a). Functional contribution of the α5 subunit to neuronal nicotinic channels expressed by chick sympathetic ganglion neurones. J Physiol.

[b49] Yu CR, Role LW (1998b). Functional contribution of the α7 subunit to multiple subtypes of nicotinic receptors in embryonic chick sympathetic neurones. J Physiol.

